# Stimuli-responsive hydrogel based on natural polymers for breast cancer

**DOI:** 10.3389/fchem.2024.1325204

**Published:** 2024-01-18

**Authors:** Khatereh Asadi, Nazafarin Samiraninezhad, Amin Reza Akbarizadeh, Abbas Amini, Ahmad Gholami

**Affiliations:** ^1^ Biotechnology Research Center, Shiraz University of Medical Sciences, Shiraz, Iran; ^2^ Department of Medical Nanotechnology, School of Advanced Medical Science and Technology, Shiraz University of Medical Sciences, Shiraz, Iran; ^3^ Guilan Road Trauma Research Center, Guilan University of Medical Sciences, Rasht, Iran; ^4^ Department of Quality Control, Faculty of Pharmacy, Shiraz University of Medical Sciences, Shiraz, Iran; ^5^ Abdullah Al Salem University (AASU), College of Engineering and Energy, Khaldiya, Kuwait; ^6^ Centre for Infrastructure Engineering, Western Sydney University, Penrith, NSW, Australia; ^7^ Department of Pharmaceutical Biotechnology, School of Pharmacy, Shiraz University of Medical Sciences, Shiraz, Iran

**Keywords:** stimuli-responsive, smart, natural polymer, hydrogel, nanogel, drug delivery, chemotherapy, breast cancer

## Abstract

**Aims:** Breast cancer is the most common malignancy among women in both high- and low-resource settings. Conventional breast cancer therapies were inefficient and had low patient compliance. Stimuli-responsive hydrogels possessing similar physicochemical features as soft tissue facilitate diagnostic and therapeutic approaches for breast cancer subtypes.

**Scope:** Polysaccharides and polypeptides are major natural polymers with unique biocompatibility, biodegradability, and feasible modification approaches utilized frequently for hydrogel fabrication. Alternating the natural polymer-based hydrogel properties in response to external stimuli such as pH, temperature, light, ultrasonic, enzyme, glucose, magnetic, redox, and electric have provided great potential for the evolution of novel drug delivery systems (DDSs) and various advanced technologies in medical applications. Stimuli-responsive hydrogels are triggered by specific cancer tissue features, promote target delivery techniques, and modify release therapeutic agents at localized sites. This narrative review presented innovation in preparing and characterizing the most common stimuli-responsive natural polymer-based hydrogels for diagnostic and therapeutic applications in the breast cancer area.

**Conclusion:** Stimuli-responsive hydrogels display bioinspiration products as DDSs for breast cancer subtypes, protect the shape of breast tissue, provide modified drug release, enhance therapeutic efficacy, and minimize chemotherapy agents’ side effects. The potential benefits of smart natural polymer-based hydrogels make them an exciting area of practice for breast cancer diagnosis and treatment.

## 1 Introduction

Breast cancer is the most common malignancy and a global public health burden. World Health Organization (WHO) reported that 2.3 million women suffer from breast cancer associated with 685,000 deaths globally at the end of 2020 (Sözen and Emir, 2023). Histopathological type, grade of tumor, and expression of specific proteins and genes are some criteria used to categorize breast cancers (Łukasiewicz et al., 2021). Breast tumors can spread to lymph nodes, lung walls, bone, the cervical region, and the brain through micrometastases (<2 mm) or macrometastases (Zhou et al., 2015). There are several primary treatments, such as surgery, radiotherapy, chemotherapy, and immunotherapy for breast cancer (Society, 2022). Conventional chemotherapy is a major adjuvant therapy that presents common complications for patients, including vomiting, nausea, fatigue, edema, myelosuppression, cognitive impairments, neurotoxicity, permanent damage to vital organs, and drug resistance (MDR). Despite significant advancements in the field of breast cancer treatment over the last decades, there is an urgent requirement for sustainable and innovative therapeutic formulations that overcome the drawbacks of current chemotherapy drugs in the advanced stages of the disease (Society, 2022).

In recent times, nanomaterials have become noteworthy in the pharmaceutical area and precision oncology due to improved pharmacokinetics by specified drug delivery systems (DDSs), modified profile release and absorption, enhanced drug bioavailability, drug plasma consistency, real-time imaging, minimal complications, and finally, treatment according to molecular features of the target tissue (Asadi and Gholami, 2021; Asadi et al., 2022). Among all the nanomaterials, hydrogels based on natural polymer with three-dimensional (3D) polymeric networks, a soft, biocompatible nature, and the ability to mimic the breast tissue microenvironment have received increased attention in cancer diagnostics and treatment. The surface modification of hydrogels based on natural polymers substantially promoted their performance and allowed them to cross biological barriers safely (Varghese et al., 2020). Moreover, natural hydrogels encompass different functional moieties such as -NH2, -COOH, -OH, and others, making them responsive to various stimuli. Since systemic DDSs remain harmless to the rest of the body, smart hydrogels hold great promise as smart materials for the local implementation of precision therapies for breast cancer (Samiraninezhad et al., 2023).

Smart or stimuli-responsive hydrogels based on natural polymers are bio-scaffolds intended for remarkable levels of control over physiochemical features. They can undergo tunable changes in swelling/deswelling capacity, permeability, porosity, network structure, flexibility, and mechanical strength (Rodríguez-Rodríguez et al., 2022). Nevertheless, such alteration induces reversible or irreversible transitions, so hydrogels can convert to their original state once the trigger has disappeared. Briefly, the hydrogels based on natural polymers act in response to various stimuli in three ways: a) providing mechanical motion (artificial tissue, soft robots) almost in combination with other nanoparticles (NPs) such as carbon nanotubes, b) regulating agent transport (on-off control of chemotropic agents release), and c) pioneering the conversion and transmission of information (targeted drug delivery, biosensors, and medical implants) (Yoshida and Okano, 2010). Stimuli-responsive hydrogels could be designed as biomimetic extracellular matrices (ECMs) with features that match those found in specific tissues (Li et al., 2023; Tian and Liu, 2023).

The breast tumor microenvironment (TME) is a highly heterogeneous ecosystem that comprises cancerous cells, different kinds of non-cancerous cells (stromal cells, epithelial cells, immune cells …), the ECM components, signaling molecules, and enzymes overexpressed in tumors (matrix metalloproteinases (MMPs) and proteolytic enzymes) (Danenberg et al., 2022). Breast tumor outgrowth demands more blood supply and nutrients. Cancerous cell supports their abnormal proliferation by induction of the angiogenesis process; new vessels are chaotic and lead to the enhanced permeability and retention (EPR) effect. Furthermore, tumor temperatures increase (37.17°C–41.44°C) due to raised microvessel density and blood flow rate (Subhan et al., 2021). High nutrient consumption in cancerous cells causes oxygen deprivation (hypoxia), glucose, and increased intra-cellular grade of reactive oxygen species (ROS).

Moreover, tumor-associated macrophages secreted tumor necrosis factor-α and induced sublethal oxidative stress (Munir et al., 2019). The glutathione (GSH) concentration in cancerous tissue was found to be as high as 1–10 mM (Argenziano et al., 2018). ROS damaged nucleotide structures and raised oxidative modified DNA base products such as 8-Hydroxy-2′-deoxyguanosine. The accumulation of 8-Hydroxy-2′-deoxyguanosine is approximately 10 times higher in breast carcinoma cells than in normal tissue from the same patient (Verigos et al., 2020). Glycolytic rates and lactic acid production increased through glucose uptake by tumor cells (the “Warburg effect”), leading to the acidic pH (typically between 5.5 and 6.9) in TME. Moreover, breast tumors reside in superficial tissue, and external stimuli such as ultrasonic, electric, and magnetic stimulation are essential to developing new therapeutic formulations based on intelligent hydrogels (Marques et al., 2021; Mehraj et al., 2021).

The current review will discuss the main features of stimuli-responsive hydrogels based on natural polymer and focus on recent research status associated with breast cancer diagnosis and treatments. We believe that the various concepts and examples collected here are essential for researchers in this field who are developing groundbreaking innovations in the future.

## 2 Natural based polymer hydrogels

Natural polymers are derived from plants, animals, and microorganisms. The main types of natural polymers include polysaccharides, proteins, polynucleotides, polyisoprenes, polyesters, and lignin (Catoira et al., 2019). Natural types are economical, readily available, and have fewer or no side effects compared to synthetic polymers. Many of these natural polymers are part of a healthy diet and have a broad scope in the recipients, drugs, implants, medical devices, food, and cosmetic industries (Morganti et al., 2023). Natural polymers are biocompatible and biodegradable with unique antioxidative, antimicrobial, and anti-inflammatory properties. In contrast, these natural materials’ main drawbacks are poor mechanical properties and exposure to the environment, altitude, humidity, availability of nutrition, and microbial contamination risk (Gholami et al., 2023). To overcome these limitations, it is essential to standardize and validate herbal production and industrial fabrication techniques (Chen, 2019).

**FIGURE 1:**
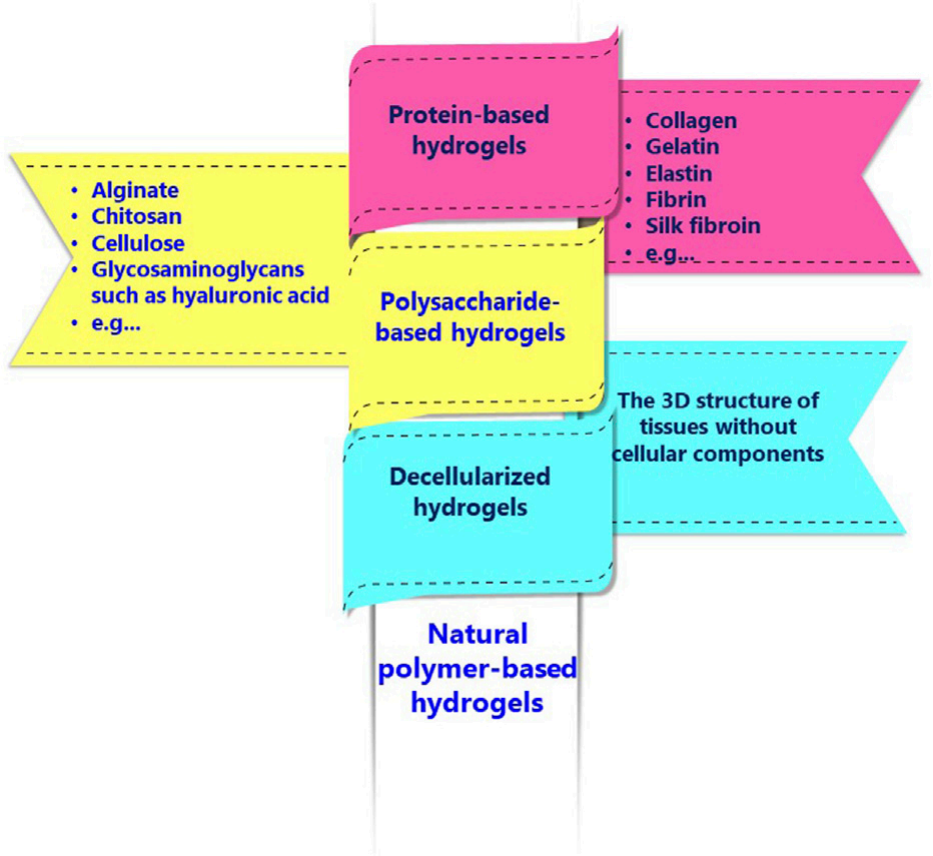
Common natural polymer-based hydrogels.

Hydrogels based on natural polymers represent significant potential in DDSs, diagnostic, regenerative medicine, and biological substitutes for artificial organs. Hydrogel is a 3D viscoelastic polymeric network that absorbs and retains significant amounts of fluids and shows similar physical properties to natural tissue (Catoira et al., 2019). Figure 1 shows typical hydrogels based on natural polymers, including protein-based, polysaccharide-based, and decellularized hydrogels.

Many crucial factors, such as mechanical parameters (tensile, flexibility, etc.), swelling–deswelling rate, stiffness, porosity, thermal stability, and degradation rate, play a role in hydrogel formulation efficacy. In addition, the hydrophilic and hydrophobic segments of polymers, the types of cross-linkers, polymer concentrations, and the synthesis route and conditions such as reaction time, temperature, pH, agitation speed, and container affect the formulation. Hydrogel categories are broad and can be described based on different aspects (Ho et al., 2022). A more applicable classification of hydrogels is mentioned in Table 1.

**TABLE 1:** Categories of hydrogels’ critical features.

Type	Subclasses	Features	Ref.
Origin	Natural	Similar to ECM, excellent biocompatibility and biodegradability	Madduma-Bandarage and Madihally (2021), Zhao et al. (2023)
	Synthetic	Excellent mechanical properties	
	Hybrid	High-water contents, high porosity, flexibility, and biocompatibility	
Polymeric composition	Homopolymers	Hydrogel network prepared from a single kind of monomer	Mahinroosta et al. (2018)
	Copolymers	Two or more different monomer types	
	Semi-interpenetrating networks	One element is a cross-linked polymer, and the other is a non-cross-linked polymer	
	Interpenetrating networks	Two independent cross-linked synthetic and/or natural polymer	
Configuration	Amorphous	No fixed shape, deliver bioactive compounds	Dong et al. (2015), Mahinroosta et al. (2018), Yu et al. (2022)
	Semi-crystalline	Display a significant Young’s modulus (up to 310 MPa) and sustain tensile stresses up to 7 MPa	
	Supermolecules	Unique physicochemical features with specific functionalities	
	Hydrocolloid aggregates	Excellent debriding and good absorption properties	
	Hydrogen bond-gels	Good mechanical properties, injectability, self-healing features, and high adaptation to irregular substrates	
Network electrical charge	Anionic	Contain negative ions	Saunders et al. (2008)
	Cationic	Contain fixed positive ions	
	Neutral	Contain the same amount of both positive and negative fixed ions	
	Ampholytic	Contain both anionic and cationic functional groups	
Size	Macrogels	The colloidal condition, physical gels (pseudo gels), chemical (true, permanent)	Lima et al. (2020)
	Microgels	High capacity for drug delivery, large surface area	
	Nanogels	Payloads locally, intracellularly, targeted drug delivery	
Physical shape	Micro/nanoparticles	Extensively encompasses microbeads and nanogels	Vasile et al. (2020)
	Film	Composites and Electrospun mates are kind of hydrogel-based films	
	Matrix	Kind of some scaffold	
	Gel	Hydrogels, injectable, and self-healing hydrogels	
Cross-linking	Physical	Synthesized by ionic interaction, crystallization, stereocomplex formation, hydrophobized polysaccharides, protein interaction, hydrogen bond	Hennink et al. (2012)
	Chemical	Synthesized by polymer-polymer conjugation, photosensitive agents, enzyme-catalyzed reaction, Schiff base reaction, epoxide coupling, addition reaction, click reaction, condensation reaction, and free radical polymerization	
Durability	Durable	Replace damaged human tissues, artificial robots	Ali et al. (2022), Chen et al. (2023)
	Degradable	Sensitive bonds can be broken due to either enzymatic or hydrolytic actions	
	Bio-degradable	Generally, natural hydrogels are bio-degradable	
Response to stimuli	Smart	Alter their properties influenced by stimuli	Bahram et al. (2016), Zhang and Huang (2021)
	Conventional	No response to internal and external stimuli, Brittle and fragile network, and cross-links	

## 3 Stimuli-responsive hydrogels based on natural polymers

**FIGURE 2:**
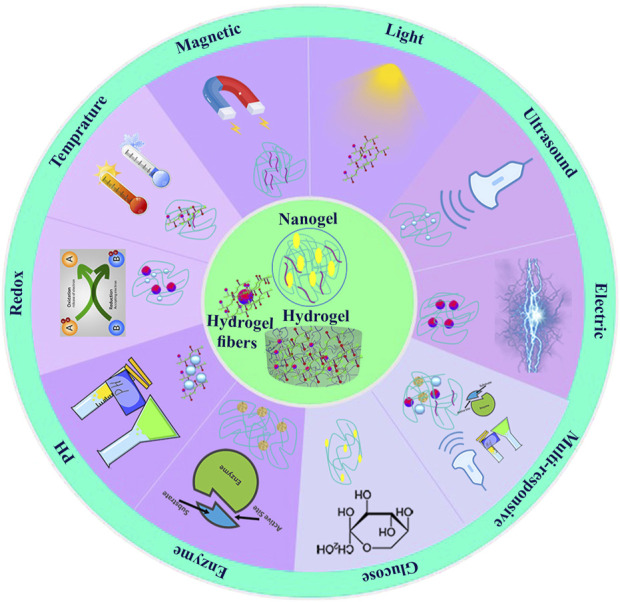
Classification of stimuli-responsive hydrogels.

The properties of stimuli-responsive hydrogels change when exposed to endogenous stimuli (produced by organisms) such as pH, temperature, glucose, enzymes, and redox, and exogenous stimuli such as radiation exposure, acoustic, magnetic force, and an electric field (Saunders et al., 2008). Fabricating effective products based on intelligent hydrogel technology requires understanding the mechanisms of natural polymers’ responsiveness to external stimuli and internal triggers (Das et al., 2020). Moreover, external stimulus-responsive hydrogels provided remote and non-invasive control (Pourjavadi et al., 2020). Stimuli-responsive hydrogels present a rich toolbox for breast tissue or intracellular compartment examination and treatments. Natural-based stimuli-responsive hydrogels are characterized by morphological, structural, mechanical, swelling, drug release, and responsive features (Yadav et al., 2022). Electron microscopy technologies were briefly used to determine morphological properties, including porosity and roughness. Nuclear magnetic resonance (NMR), Fourier transform infrared spectroscopy (FTIR), and X-ray photoelectron spectroscopy (XPS) were applied for structural composition assessment. The swelling behaviors were evaluated through changes in volume or weight. The rheometer provides information about viscoelastic properties, while tensile and other mechanical tests provide insight into hydrogels’ mechanical and degradation characteristics (Rezaei et al., 2021; Gul et al., 2022). Figure 2 displays a brief insight into the stimulus-responsive hydrogels.

### 3.1 PH-responsive hydrogels based on natural polymers

The physiological pH of the majority of normal human tissues and blood ranges from 7.1 to 7.45, with a few exceptions, such as the vagina, skin, and gastrointestinal tract, and the lysosomes, Golgi, and endosomes, which range from 1.0 to 7.0. Pathological conditions are often associated with remarkable pH changes and aid in targeting DDSs based on stimulus-responsive strategies (Lee and Shanti, 2021). Breast cancerous cells, due to their high glycolysis rates and increased metabolism rates, reduced extracellular pH values to an acidic state (Gao et al., 2020). Natural polymers with ionizable networks (pKa values from 3 to 10) are considered suitable candidates for planned biocompatible chemotherapy agent DDSs, especially when they contain pH-responsive hydrogels (Karimi et al., 2016). These pH-responsive products can accept or donate protons and have altered hydrophilicity in breast tumor environments. Natural polymers, whether utilized as monomers or copolymer systems containing other acid-functionalized groups or polymers triggered by pH stimuli, exhibit enhanced mechanical properties and undergo conformational variations in response to environmental pH changes, thereby facilitating the release of chemotherapeutics (Abdouss et al., 2023).

PH-responsive hydrogels are designed based on two main strategies: conformational and charge-shifting in response to pH variation (cationic and anionic) and acid-cleavable bonds into the polymeric backbone. These hydrogels facilitate cargo release upon interaction with targeted organelles, ligands, or charge modifications of the network (Rezk et al., 2019; Yadav et al., 2022).

**FIGURE 3:**
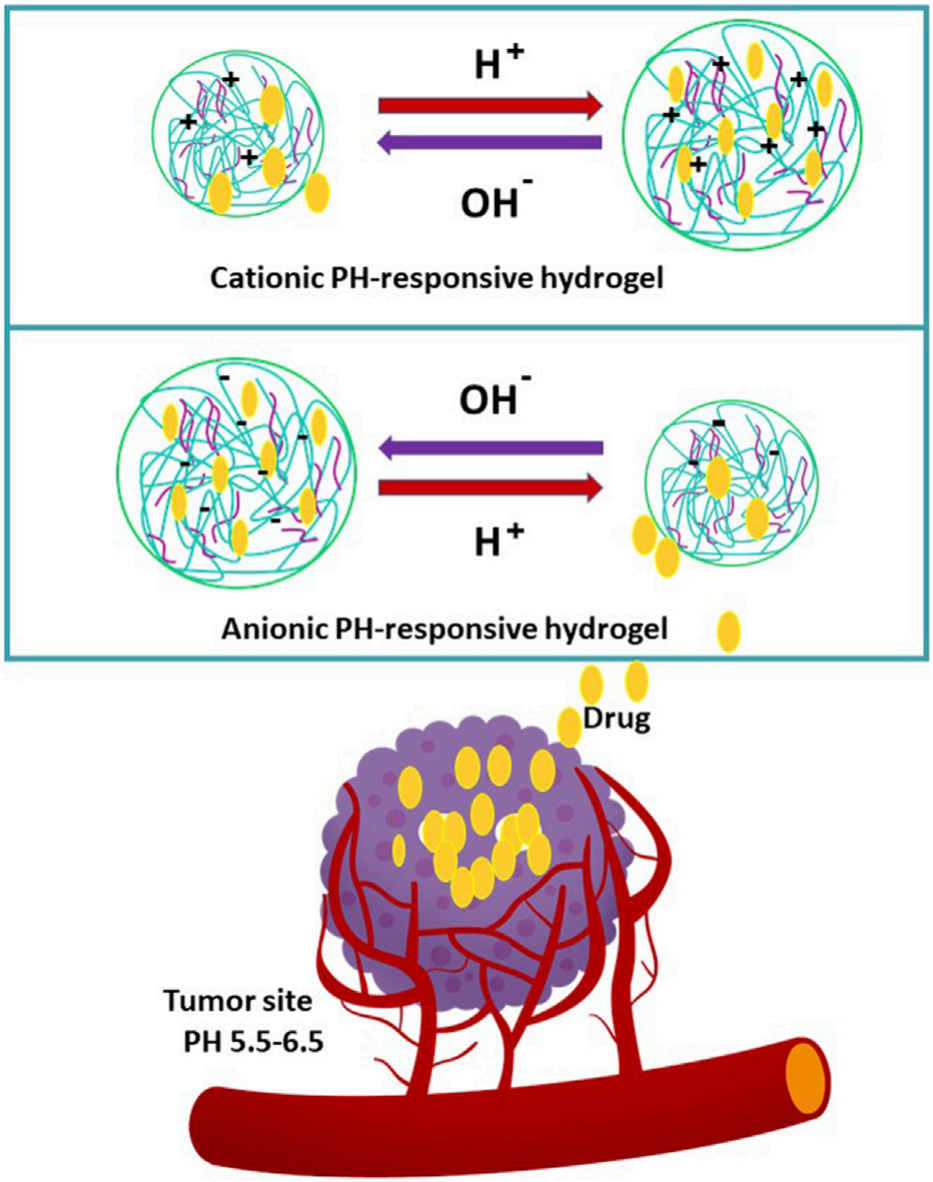
Fundamental of cationic and anionic pH-sensitive hydrogels.

Cationic and anionic pH-sensitive hydrogel approaches are displayed in Figure 3. Polymers such as albumin and cellulose, which possess a weakly acidic group, expand and swell at a basic pH (anionic), whereas those with an alkaline group (such as amine) attached to the hydrophobic backbone exhibit swelling at an acidic pH (cationic). For instance, the tumor environment’s pH is lower than the pKa of a cationic polymer, and alkaline groups (e.g., amine) are positively charged (NH3+) by protonation. The hydrogel network has been expanding and swelling upon electrostatic repulsion between polymer charges, facilitating cargo diffusion (Yadav et al., 2022).

An alternative approach involved the conjugation of acid-labile bonds into the natural polymeric networks, which are cleaved in the acidic condition of ECM after endocytosis in tumor cell endosomes or lysosomes. For instance, during normal physiological pH conditions, hydrazine linkage is very stable, but it cleaves around the tumors or subcellular components in an acidic state. Other pH-liable linkers are the acetal bonds and cis-aconityl groups of the maleic acid amides (MAA) (Liu et al., 2017).

Recently, many researchers have studied natural pH-stimuli-responsive hydrogels in breast cancer chemotherapy areas. Abdouss et al. created a novel pH-sensitive nanogel based on chitosan (CS)-loaded curcumin (CUR) that was fabricated via water/oil/water (W/O/W) emulsification methods. Graphene nanosheets (g-C3N4) were fabricated through a simple one-step pyrolysis process using thiourea as a precursor and entrapped into CS polyacrylic acid (PAA) nanogel. For nanogel preparation, PAA powder (0.5 g) was combined with a homogeneous CS (2%, w/v) solution to obtain the hydrogel. Then, g-C3N4 NPs (0.1%, w/v) were mixed under stirring until a homogeneous solution was attained. For the drug loading step, CUR (5 μg/mL) was added to the CS/PAA/gC3N4 mixture dropwise under the heater stirrer for half an hour. Finally, CUR was loaded into CS/PAA/gC3N4 nanogel and prepared. For double emulsion synthesis, span-80 surfactant (0.2%, v/v) was added to the nanogel dropwise under stirring to prepare a span-80-coated CS/PAA/g-C3N4/CUR. Then, the solution (10 mL) was added to 30 mL of hazelnut oil (the oil phase) dropwise under a magnetic stirrer. Then, the CUR-loaded nanogels separate from the hydrophobic phase. The mixture was kept without agitation to separate the hydrophobic and hydrophilic phases. After removing the oil phase and centrifuging at 4,500 rpm for 15 min, pH-stimuli-responsive nanogels were obtained. A freeze dryer is used to produce a homogeneous powder. Release profiles of CUR-loaded CS/PAA/g-C3N4 nanogels were assessed at both acidic (5.4) and neutral (7.4), with the result proving high CUR loading and an excellently controlled pH-sensitive release profile. CUR-loaded pH-stimuli-responsive nanogels lead to the highest rate of apoptosis and cell viability (less than 50%) in breast adenocarcinoma (MCF-7) cells (Abdouss et al., 2023).

Cimen et al. designed a pH-responsive hybrid hydrogel via the formation of an acid-cleavable bond (hydrazine) among hydrazide-modified gelatin (Gel-ADH) and aldehyde-polyethylene glycol (diBA-PEG) polymers. Doxorubicin (DOX) loaded laponite (LAP) during gelation, yielding hybrid Gel-ADH/diBA-PEG/LAP@DOX hydrogels. The gel−sol transition behaviors of hybrid pH-stimuli-responsive hydrogel indicated significant gelation stability, which led to a controlled and pH-dependent long-term drug release profile. The cytocompatibility of hybrid Gel-ADH/diBA-PEG/LAP@DOX hydrogels on the two normal cell lines, such as those transfected with SV40 (SVCT) and human umbilical vein endothelial cells (HUVEC), showed significant cytocompatibility and supported cell proliferation. Furthermore, the MTT result of the hybrid smart hydrogel on the MCF-7 and triple-negative breast cancer cell line (MDA-MB-231) offers its potential as an outstanding chemotherapeutic DDS for long-term and controlled release treatment for breast cancer (Cimen et al., 2021).

In another study, *in vivo* release studies were conducted on albino rabbits. The study focused on the Theobid SR tablet from Cipla (product A) and theophylline-loaded hydrogels of CMC, both containing 300 mg of theophylline. Blood samples were withdrawn at different intervals to estimate plasma concentrations of theophylline. The results showed that product A had a peak plasma concentration (Cmax) of 12.34 ± 2.42 μg/mL after oral administration. In contrast, product B had a Cmax of 09.69 ± 4.12 μg/mL, indicating lower plasma concentrations for product B. Both products maintained the therapeutic concentration range of theophylline for about 24 h after a single oral dose. The time taken to reach Cmax (Tmax) was 5.0 ± 0.81 h for product A and 6.0 ± 0.75 h for product B. Product B also exhibited a lower Cmax and prolonged Tmax, suggesting slow drug release and prolonged and controlled *in vivo* delivery. These findings align with the *in vitro* drug release rate from the hydrogel.

A recent study evaluated the anti-tumor efficacy of a paclitaxel (PTX) drug delivery system using a pH-responsive hydrogel. The *in vivo* study on the mice model confirmed the remarkable anti-tumor effectiveness of the PTX drug delivery system using the pH-responsive hydrogel in H22 tumor-bearing mice. It exhibited superior tumor growth inhibition compared to normal saline and free paclitaxel solution after 3 days of intratumoral administration. It demonstrated a significant reduction in tumor size and minimal toxicity. Tumor excision and immunohistochemical analysis further supported the findings (Raza et al., 2019).

PH-responsive hydrogels face the disadvantage of having unpredictable behavior in terms of turning on or off in the tissue.

### 3.2 Glucose-responsive hydrogels based on natural polymers

A glucose-responsive hydrogel responds to changes in glucose levels in the surrounding environment (Walter et al., 2019). These hydrogels incorporate a receptor, often based on boronic acid, which can selectively bind to glucose molecules (James and Schrader, 2007). When glucose interacts with the receptor, it triggers a physical response in the hydrogel, such as swelling or a volume change (Walter et al., 2019). This response serves as a sensor signal and can be quantitatively analyzed to determine glucose concentrations, particularly under physiological conditions (Matsumoto et al., 2010).

Hao et al. developed a near-infrared (NIR) laser and glucose-responsive hydrogel for breast cancer treatment. First, iron dichloride tetrahydrate (FeCl24H2O) and polyvinylpyrrolidone were added to degas deionized water and stirred at room temperature. Next, gallic acid (GA) was dropwise added to the above solution and stirred in the nitrogen atmosphere. The obtained GAFe nanocomplexes were condensed and purified. The hydrogel was fabricated by blending N, N-dimethyl acrylamide (DMAA), polyethylene glycol double acrylates (PEGDA), GA-Fe nano complexes, and glucose oxide (GOx). To assess the impact of the glucose mass and NIR radiation on gelation, different glucose concentrations (0.2, 0.5, and 1 mg/mL) were mixed with the components and subjected to two gelation conditions: incubation at room temperature and exposure to 808 nm laser irradiation (Hao et al., 2020).

The photothermal properties of the GA-Fe nano complexes demonstrated their ability to increase temperature rapidly when exposed to an 808 nm laser. They maintained their performance after multiple laser irradiation cycles. The nano complexes were found to be efficient Fenton catalysts since they degraded methylene blue in the presence of varying H_2_O_2_ concentrations. Their catalytic activity was temperature-dependent and enhanced by NIR irradiation. GOx, a stable enzyme capable of generating H_2_O_2_ from glucose, exhibited optimal catalytic activity at specific temperatures. When combined with the GA-Fe nano complexes, a cascade reaction was observed that produced highly reactive •OH radicals. This reaction was glucose-dependent and temperature-sensitive, with the highest efficiency achieved at 45°C and under NIR radiation. The cytotoxicity of the GOx-GA-Fe catalyst couple was assessed on breast cancer cells (4T1 murine breast cancer cells, MCF-7, BT474, SK-BR-3, and MDA-MB-231 human breast cells) and found to induce oxidative stress and cell death, particularly in the presence of glucose. This cytotoxicity was selective for cancer cells and had minimal impact on normal cells.

Moreover, NIR exposure significantly enhanced the toxicity of the catalyst couple, making it glucose- and NIR-responsive. To minimize side effects, stimuli-responsive hydrogel systems based on natural polymers with excellent biocompatibility are promising to inject the GOx-GA-Fe nano complexes directly into the intratumoral region with negligible invasiveness. Moreover, hydrogel hydration properties reduce side effects (Hao et al., 2020).

*In vivo* studies on female mice established a successful orthotopic breast tumor model. Early therapeutic efficacy of various treatments was assessed through histological analyses at 24 h post-treatment. Using a ROS probe, they further investigated the hydrogels’ impact on intra-tumoral oxidative stress amplification. Intra-tumoral administration of the catalyst couple combined with NIR radiation enhanced oxidative stress within the tumor. The potency of the catalyst couple was determined in an orthotopic breast tumor model. Treatment involving intra-tumoral administration of the catalyst coupled with NIR radiation demonstrated the most effective tumor inhibition. The results of tumor growth and wet weighting data approved that. Histological examination also revealed severe tumor damage and cell apoptosis (Hao et al., 2020).

Developing a glucose-responsive system faces several challenges, including improving responsiveness at physiological pH, inactivity of glucose oxidase from high temperature, pH changes, and enzymatic action. Inflammation caused by hydrogen peroxide is also believed to be an issue for GOx-based smart delivery systems (Roy et al., 2022).

### 3.3 Redox-responsive hydrogels based on natural polymers

Redox-responsive hydrogels exploit the distinctive reducing environment of tumor cells characterized by high levels of GSH. GSH regulates the cellular reducing environment by influencing disulfide bond formation and fragmentation, which makes it an ideal trigger for cargo release in the redox-responsive system (Aon et al., 2010; Such et al., 2015). These systems offer stability in normal tissues, a rapid response to elevated GSH concentrations in tumor cells, and the potential for enhanced therapeutic effects upon cytoplasmic release. Redox-responsive nanocarriers are categorized into those with disulfide bonds, di-selenide bonds, and other structures sensitive to reducing environments (Beld et al., 2007; Ji et al., 2014).

**FIGURE 4:**
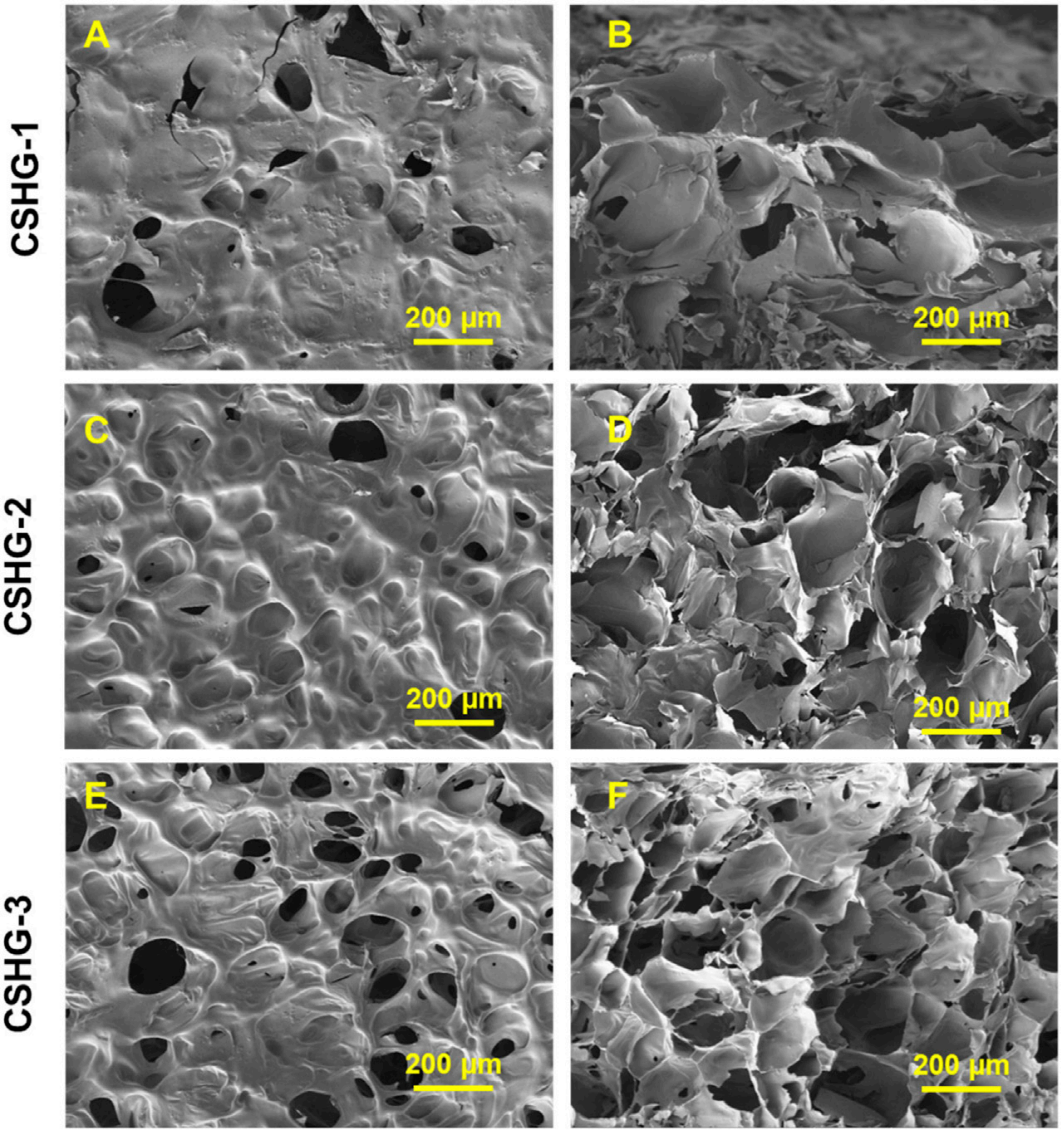
SEM images **(A–F)** of a redox-responsive hydrogel’s surface, porosity, and cross-sectional area based on chitosan polymer (Vu et al., 2023).

Figure 4 presents a redox-responsive hydrogel based on the chitosan polymer’s surface, porosity, and cross-sectional area. In this study, redox-responsive hydrogels were fabricated by performing the inverse electron demand Diels–Alder (IEDDA) reaction, which conducted the “click” reaction among a norbornene (Nb)-substituted CS (CS-Nb) and polyethylene glycol (PEG)-type disulfide as a water-soluble disulfide cross-linker (Nb and tartrazine (Tz) in aqueous solution). The Click chemistry offers significant advantages in creating redox-responsive hydrogels with a higher surface and porosity for improved GSH (reducing agent) functions. A polyethylene glycol (PEG)-type disulfide cross-linker is required to design water-soluble, reduction-responsive, non-toxic hydrogels. (Vu et al., 2023).

Wang et al. designed a novel formulation based on hyaluronic acid (HA) for targeted DDSs and photodynamic therapy against breast cancer (Wang R. et al., 2021). Blank NPs were formed by dissolving 3 mg of the polymer HA-cysteamine-docosahexaenoic acid/chlorin e6 (CHD) in 1.8 mL of phosphate-buffered saline (PBS) with a pH of 7.4 and subjecting the solution to probe sonication, followed by filtration. Then, 7.5 mg of CHD was dissolved in 2.5 mL of PBS, incrementing DTX with agitation, applying ultrasound, and conducting probe sonication to produce DTX/CHD. The resulting solution was dialyzed, centrifuged, and filtered to obtain DTX and CHD NPs. The NPs incorporated HA and docosahexaenoic acid (DHA) for potential use in mono-photodynamic therapy (Wang R. et al., 2021).

In the redox-responsive behavior investigation, di-thiothreitol (DTT) exposure caused the particle size of DTX and CHD NPs to increase. A shift from unimodal to bimodal size distribution occurred due to disulfide bond breakage, separating hydrophobic and hydrophilic segments within the polymer, which resulted in larger particles with varied sizes (Wang R. et al., 2021). Fluorescence recovery analysis indicated that chlorin e6 (Ce6) aggregated within NPs’ hydrophobic core when connected to CHD polymer, which led to concentration quenching and reduced fluorescence. However, after DTT exposure, a fracture of the disulfide bond occurred, freeing Ce6 from the polymer, reducing concentration quenching, and causing fluorescence recovery (Hou et al., 2016; Zhu et al., 2020). This phenomenon supports the potential for strong fluorescence imaging of NPs in reduced tumor microenvironments.

Regarding singlet oxygen production under reduction conditions, DTX/CHD NPs exhibited weaker production than free Ce6 under NIR irradiation. However, DTT exposure improved the ability of CHD NPs to generate reactive oxygen species due to changes in the structure and fluorescence resonance energy transfer (FRET) effects (Zeng et al., 2016; Wang R. et al., 2021). This effect indicated that the NPs’ structure was disrupted in the tumor’s reduced environment, leading to increased reactive oxygen production. Additionally, drug release studies evaluated DTX release from DTX/CHD NPs under varying conditions. DTX release was significantly faster in a high-reducing environment (20 mM, DTT) than in low-reducing conditions (20 μM, DTT). The initial burst release of DTX from NPs was attributed to surface adsorption and rapid dissolution in the release medium (Wang R. et al., 2021).

The *in-vivo* evaluations in mice revealed that CHD NPs accumulated more in tumors and exhibited sustained presence, while free Ce6 was quickly cleared. Regarding anti-tumor efficacy, DTX/CHD plus near-infrared treatment showed the most potent effect, significantly slowing tumor growth compared to single chemotherapy or photodynamic therapy. This effect was confirmed by tumor volume, weight, and histological analysis, indicating extensive cell destruction and apoptosis (Wang R. et al., 2021).

Nieto et al. prepared gellan gum hydrogels as paclitaxel carriers for human epidermal growth factor receptor 2 (HER2) positive breast cancer therapy (Nieto et al., 2022). Gellan gum (1.5%, w/v) was dissolved in acetate buffer and PBS. After achieving uniform solutions, the temperature was reduced. Subsequently, 1-ethyl-3-(3-dimethylaminopropyl) carbodiimide and N-hydroxy succinimide solutions were successively introduced at a 1:50 (v/v) ratio. After a brief stirring period, L-cysteine solutions with varying concentrations (1.5 mg/mL, 3 mg/mL, and 4.5 mg/mL) were added at a 1:50 (v/v) ratio to achieve distinct levels of chemical cross-linking. The solutions underwent gelation at room temperature throughout the night to form gellan gum hydrogel (Nieto et al., 2022).

The viscoelastic properties were evaluated using dynamic oscillatory frequency sweep assays. The frequency sweep data indicated that all samples exhibited typical gel behavior, as the storage modulus was at least ten times higher than the loss modulus. Additionally, both storage and loss moduli were nearly independent of frequency, characteristic of entangled gels. However, storage modulus values were higher when hydrogels were prepared in PBS than in acetate buffer. This result indicated that hydrogels prepared in PBS formed gels more rapidly and had a higher viscosity. This result was expected since PBS contains K^+^ and higher Na^+^ concentrations, which promote greater cross-linking. Increasing the L-cysteine concentration led to higher storage modulus values, indicating the formation of more robust 3D networks due to increased chemical cross-linking. The reduction in storage modulus was less noticeable when hydrogels were disulfide cross-linked with higher L-cysteine concentrations or when they were synthesized in PBS instead of acetate buffer. Swelling kinetics were analyzed by immersing the hydrogel samples in water, NaCl solutions, PBS, DMEM, and buffers to mimic physiological conditions. Most hydrogel samples reached equilibrium after approximately 240 min of soaking in the different media. The degree of cross-linking is inversely correlated with swelling capacity, as higher L-cysteine concentrations reduce pore size and the volume absorbed by the hydrogels. Gellan gum hydrogels prepared in acetate buffer exhibited greater swelling capacity, especially in alkaline media. The thermal was evaluated, and all hydrogels exhibited a two-step weight loss, with the first step attributed to the evaporation of adsorbed buffer/H2O in the samples. The second step indicated polymer degradation and hydrogel network destruction. Higher degrees of cross-linking resulted in improved thermal stability. The study showed that PTX release was controlled, with a slight initial burst release. Hydrogels with higher cross-linking exhibited a slower PTX release. The release was more pronounced in acidic conditions and could be accelerated in the presence of high glutathione concentrations, indicating redox-responsive properties conferred by L-cysteine-based cross-linking (Nieto et al., 2022).

The anti-tumor activity of hydrogel was assessed *in vitro* on HER2-overexpressing breast carcinoma cell lines. The results indicated that PTX-loaded Gellan gum hydrogels effectively reduced cell viability over time. Live/dead staining and lactate dehydrogenase leakage assays supported these findings, confirming the impact of Gellan gum hydrogel-based drug delivery on cell membrane integrity (Nieto et al., 2022).

### 3.4 Enzyme-responsive hydrogels based on natural polymers

**FIGURE 5:**
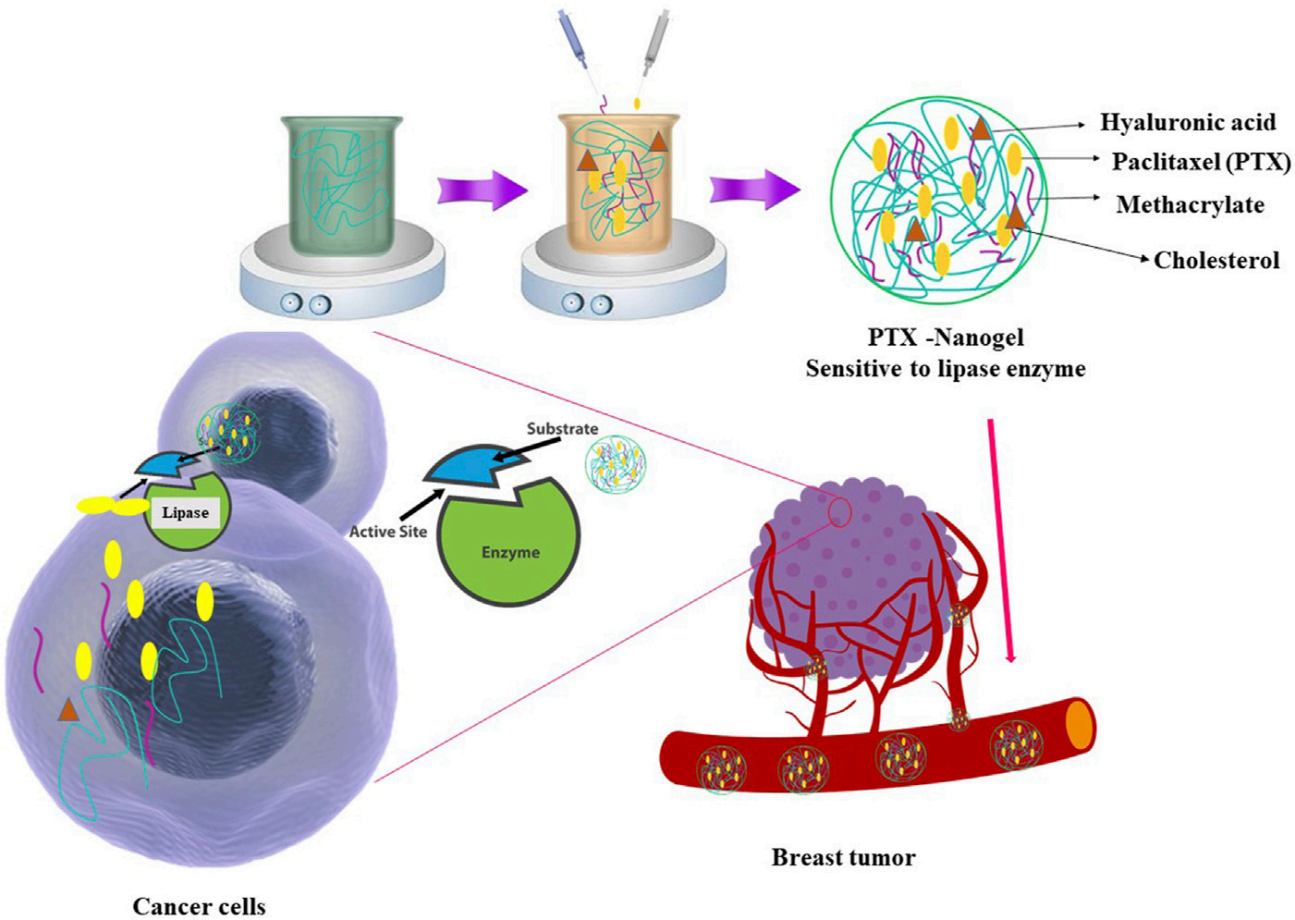
The design of enzymatic-responsive hydrogel based on their structural elements.

In the framework of controlled DDSs, enzyme-responsive hydrogels based on natural polymers stand out as a remarkable category among stimulus-responsive biomaterials (Figure 5). Compared to normal tissues, the high concentration of enzymes in cancerous lesions makes them ideal triggers for precise drug delivery (Stern and Jedrzejas, 2006; Wei et al., 2018). Various enzymes, such as protease, lipase, oxidoreductase, and hyaluronidase, have been used to trigger drug release. Hyaluronidase, in particular, is highly expressed in the microenvironment of various tumors as it plays a pivotal role in the degradation of HA, a fundamental component of the extracellular matrix (Stern and Jedrzejas, 2006). As a result, HA-based formulations exhibit hyaluronidase-responsive drug release.

Gao et al. constructed an enzyme-sensitive paclitaxel-loaded HA nanogel to treat breast cancer (Gao et al., 2022). Methacrylated HA (MHA) was created by modifying HA with methacrylic anhydride, while cholesteryl-2-aminoethyl carbamate (CHOL-NH2) was altered by adding cholesteryl and ethylenediamine. Subsequently, cholesterol-grafted methacrylated HA (CMHA) was prepared by activating MHA and adding CHOL-NH2. Nanogels were formed through radical polymerization of CMHA with diethylene glycol diacrylate and tetramethyl-ethylenediamine, followed by dialysis. Finally, biotin-modified nanogels (Bio-NG) were obtained by modifying the nanogel with biotin after activating it with carbodiimide, followed by dialysis (Gao et al., 2022).

PTX was successfully loaded into nanogels without altering their physical properties. The nanogels showed sensitivity to enzymes like hyaluronidase and lipase, leading to their degradation and enzyme-triggered PTX release. The results of the cellular uptake assay have demonstrated that biotin-modified nanogels (Bio-NG) were taken up more effectively by cells because of receptor-specific interactions. Subcellular localization tests have revealed that the nanogels were transported to lysosomes within cells, where they gradually destabilized and released their cargo into the cytoplasm (Gao et al., 2022).

The ability of nanogels to kill breast cancer tumors was assessed in an *in vivo* study. Bio-NG showed superior tumor targeting compared to control nanogels. Pharmacokinetic evaluation revealed that PTX-loaded nanogels had extended circulation times, higher drug exposure, and slower clearance compared to Taxol. PTX/Bio-NG demonstrated the highest tumor inhibition rate (94%), followed by PTX/NG (73%) and Taxol (59%). The mouse’s body weight remained stable throughout the treatment, indicating safety. No deaths were observed in the PTX/Bio-NG and PTX/NG groups, confirming efficacy and safety. Histological analysis showed that the PTX/Bio-NG group had less tumor necrosis and that major organs remained unaffected (Gao et al., 2022).

Aslzad et al. developed an efficient enzyme-responsive carrier for DOX delivery composed of a CS/gelatin hybrid nanogel containing gold NPs (CS/AuNPs/Gel) (Aslzad et al., 2023). CS/AuNPs were synthesized by dissolving CS in 1% acetic acid and adding HAuCl4 (36 mM) while stirring at 70°C, resulting in a color change to red-purple. CS/AuNPs/Gel nanogels were made by stirring a mixture of CS/AuNPs and Gel (1%, w/v) at room temperature for 30 min. Then, tripolyphosphate (TPP) solution (0.5%, w/v) was added dropwise to form CS nanogels through ionic cross-linking. For DOX incorporation in S/AuNPs/Gel nanogels (CS/AuNPs/Gel-DOX), DOX was added before the TPP addition and stirred for 30 min (Aslzad et al., 2023).

The result of the cellular uptake test showed that CS, AuNPs, and Gel-DOX nanogels were efficiently internalized by MCF-7 cells because of their positive charge and small size. The *in vitro* evaluation of the drug’s kinetics revealed that, even with the addition of gelatin, the drug’s kinetics were limited within 5 hours at pH levels of 7.4 and 5.7 (14.3% and 13.6%, respectively). In the presence of gelatinase, a more rapid release (20.1% and 21.65% within 5 h) occurred, surpassing the release without the enzyme after 24 h. Equilibrium drug release reached 71.3% and 62.35% after 48 h at pH 7.4 and 5.8, respectively, with lower release in samples without enzymes (27.5% and 50.8%). This suggests that CS, AuNPs, and Gel-DOX nanogels remain stable in biological conditions but promote drug release through enzymatic degradation for effective tumor treatment (Aslzad et al., 2023).

### 3.5 Temperature-responsive hydrogels based on natural polymers

Temperature-responsive hydrogels based on natural polymers have been broadly applied for breast cancer therapy. The drug delivery rate of the thermo-responsive hydrogel at the target site depends on the tumor microenvironment’s elevated temperature (37.17°C–41.44°C) (Farjadian et al., 2019). Creating thermo-responsive hydrogels requires adding polymers or functional moieties sensitive to minor temperature variations compared to normal physiological body temperature (37°C) (Qu et al., 2015). A thermo-responsive hydrogel was developed based on the critical solution temperature (CST), at which polymers undergo a sol-gel phase transition. The temperature-responsive hydrogels are often divided into two main categories: polymers with a lower critical state temperature (LCST), which cause the constitution of hydrogen bonds and swell at temperatures below the LCST (negative temperature sensitivity), and polymers with above the upper critical state temperature (UCST), which swell at temperatures above UCST (positive temperature-sensitive polymers). Natural polymers with LCST, such as CS, have a phase transition temperature near body temperature and are significantly used for thermo-responsive hydrogels (Marques et al., 2021; Yeo and Park, 2021).

For example, Fathi et al. developed DOX loaded in dual thermo- and pH-responsive hydrogels with potential breast cancer therapy. The stimuli-responsive hydrogel was synthesized with CS, poly-nisopropylacrylamide-co-itaconic acid (PNIAAm-co-IA), and glycerophosphate (GP) through the ionic cross-linking method. The CS solution (2%, w/v) was combined with an appropriate amount of PNIPAAm-co-IA and stirred until the complete dissolution of the copolymer occurred. This step adds DOX (3 and 0.1 mg per mL of hydrogel) to the copolymer mixture. Then, the aqueous solution of GP (40%, w/v) was added dropwise to the mix under stirring at pH 7.4. The cell viability assay showed that the stimulus-responsive hydrogel is biocompatible. The proliferation of MCF-7 cells cultured on the DOX-loaded stimuli-responsive hydrogel was examined by 4′,6-diamidino-2-phenylindole (DAPI) staining, which further approved their potential for breast cancer treatment (Fathi et al., 2019). Since the body temperature changes easily following physical exercise, the behavior of the thermosensitive hydrogels can be very challenging.

### 3.6 Light-responsive hydrogels based on natural polymers

Light-responsive hydrogels display alteration in their properties through heat transfer, charge change, and photo-isomerization upon exposure to (NIR), ultraviolet (UV), and visible light. Light exposure leads to precise and substantial alternation of the hydrogel network at a wireless system without the necessity of contact or proximity (Wang H. et al., 2021). Furthermore, photosensitive hydrogels provide an on/off control release manner, photothermal therapy, and biosensoring system for breast cancer. The drawbacks of visible (limited penetration into tissue depth) and UV radiation (low penetration and risk of damaging tissues) prevented them from being suitable for clinical purposes. In contrast, NIR exposure with a wavelength range from 650 to 950 nm is considered a safe and strengthened tissue penetration light source for planned chemotherapeutic DDSs (Algorri et al., 2021). The incorporation of photoactive moieties, such as nitrobenzyl ester linkers, spiropyrans, and azo groups, into the natural polymeric backbone or photosensitive NPs or agents has aided in the development of light-stimulating hydrogels (Luo et al., 2019; Xing et al., 2022).

For instance, an *in vivo* study was conducted on 3D printed polydopamine (PDA) and alginate (Alg) core-shell NIR-triggered hydrogel fibers and scaffolds containing DOX for the treatment of residual breast cancer and prevention of local recurrence after surgery. Firstly, the printing inks were prepared from PDA (0.5%, w/v) and Alg (15.3%, w/w) and loaded into two printing tubes. DOX-loaded (1%, w/w) NIR-sensitive hydrogels as the core part were co-injected and coaxially 3D printed into core-shell hydrogel scaffolds. PDA displayed a significant photothermal effect under NIR exposure, which could elevate the temperature, induce the gel-sol transition, and subsequently result in the DOX release from the loosened light-responsive hydrogel network. Thus, a NIR-triggered polydopamine (PDA)/Alg core-shell hydrogel scaffold could be a biomimetic implant that fills the cavity with breast tissues after surgical resection, eliminates residual cancerous cells, and prevents the recurrence of malignancy (Wei et al., 2020). However, the carcinogenic potential and poor penetration depth of electromagnetic waves in breast tissue confined the usage of this kind of smart hydrogel. Furthermore, photosensitive agents are commonly toxic, and standard chemical engineering is required for safe and effective smart hydrogel developments.

### 3.7 Ultrasonic-responsive hydrogels based on natural polymers

Ultrasound has been extensively applied as a non-ionizing, non-invasive diagnostic examination for breast diseases. The Food and Drug Administration (FDA) has established safety guidelines based on a mechanical index, a thermal index, and other critical factors for the maximum allowed ultrasound dose on humans. In the past decade, it has been extensively used in the ablation of tumors, facilitating DDSs, enhancing absorption in cells and tissues, secreting signaling molecules, and influencing chemical and biological processes (Farrell et al., 2021). Ultrasound-responsive hydrogel platforms based on natural polymers allow localized, site-controlled drug delivery, deep tissue penetration, spatiotemporal control, and real-time imaging. The ultrasonic-responsive hydrogel DDSs aid in direct drug diffusion into the cancer tissues and are supported by nanocarriers for enhanced retention time and precise drug delivery. Ultrasound-triggered hydrogels are based on the amount of energy divided into high-intensity focused ultrasound (HIFU) and low-intensity focused ultrasound (LIFU) (Baghbani et al., 2017). Polymeric construction, cross-linker, molecular assembly, fabrication method, and other physicochemical aspects determined the ultrasound-responsive hydrogel properties. For instance, weaker bonds break quickly and require low-intensity ultrasound energy (Marques et al., 2021). The intensity of responsiveness, mechanical force location, and ultrasound-responsive hydrogels’ mechanochemical activity depends on these key factors, which will regulate the DDSs’ efficacy. Many novel ultrasound-responsive hydrogels based on natural polymers have been developed until now (Huebsch et al., 2014).

For example, a study developed an ultrasound-responsive hydrogel composed of a fibrin matrix to explore the effects of local mechanical properties on triple-negative breast cancer cell signaling. They assumed that cancer cells continually sense and alter intracellular signaling cascades to respond to ultrasound waves. The ultrasound-responsive hydrogel was fabricated by casting fibrin (10 mg/mL), aprotinin (0.05 U/mL), phase-shift emulsion (0.01%, v/v), and thrombin (2 U/mL) followed by polymerization at room temperature. Treatment of ultrasound-responsive hydrogel with focused ultrasound drives acoustic droplet vaporization (ADV) in a spatiotemporally controlled manner, inducing local compaction and stiffening of the fibrin matrix adjacent to the matrix-bubble interface and providing live single-cell imaging of the MDA-MB-231 breast cancer cell line. The final result revealed that two crucial kinases involved in cancer progression, such as protein kinase B (also known as Akt) and extracellular signal-regulated kinase (ERK), stimulated basal and growth factors and exhibited a correlation with the intensity of ADV-induced bubbles both *in vitro* and in a mouse model. Thus, ultrasound waves induced local alteration in ECM compaction, which regulates breast cancer’s Akt and ERK signaling pathways. The novel ultrasound-responsive fibrin hydrogel technology could visualize ECM mechanics in cell signaling and breast cancer biology (Humphries et al., 2022).

Yu Lee et al. developed ultrasound-responsive nanogels with a combination of CS and deoxycholic acid containing perfluoropentane and iron oxide that entrapped small interfering RNA (siRNA) for breast cancer cells. The mean hydrodynamic diameter of the ultrasound-responsive CS nanogels (CNDs) in deionized water was 257.6 ± 10.9 nm. However, upon exposure to ultrasound (1.8 MHz and 335 kPa peak negative pressure) for 45 s and perfluoropentane (PFP), the phase change from liquid to gas increased to 3822.2 + 226.4 nm. The findings demonstrate that ultrasound exposure did not adversely affect siRNA functionality and that the ultrasound-responsive nanogels significantly enhanced siRNA uptake, resulting in a high rate of breast cancer cell apoptosis (52.4%) after ultrasound treatment (Lee et al., 2017). However, ultrasound waves might damage the breast tissue temporally, but repetitive administration is associated with irreversible injuries. The piezoelectric agents and other ultrasonic responsive reagents could associated with complication and safety concerns that expected the surface modification and chemical engineering to remove the clinical application obstacles.

### 3.8 Electric-responsive hydrogels based on natural polymers

Among the stimuli to which the smart hydrogels respond, electrical fields offer distinct advantages due to their precise controllability, even in wireless devices. Electrically conductive polymers can manipulate cellular functions, particularly in muscular, neuronal cells, and superficial mammary tissue (Wang et al., 2015; L et al., 2014). Electrically conductive polymers can undergo oxidation or reduction processes to release incorporated small molecules, making them a desirable approach for on-demand DDSs (Alshammary et al., 2016; Saman et al., 2016).

Qu et al. fabricated a conductive hydrogel for localized drug release (Qu et al., 2019). Aniline trimer (AT) was prepared by dissolving N-phenyl-1,4-phenylenediamine in an aqueous mixture of acetone and acid and adding ammonium persulfate dropwise. Then, dextran/hexamethylene diisocyanate (Dex/HDI) hydrogels were formed by cross-linking dextran with HDI in dry DMSO. Conductive Dex-AT/HDI hydrogels were synthesized with the addition of AT. Dexamethasone and indomethacin loaded in Dex-AT/HDI conductive hydrogel (Qu et al., 2019).

The hydrogels demonstrated enhanced mechanical properties, with significant modulus values ranging from 1,650 to 2,450 Pa, and their cross-linking density decreased as AT content increased. Dex-AT/HDI hydrogels had a higher swelling ratio than Dex/HDI hydrogels, and pore sizes correlated with AT content. Higher AT content led to larger pore sizes. The conductivity of hydrogels increased as the AT content increased. Release kinetic showed that AT content affects drug amounts, and higher AT leads to an increased release rate. The hydrogels also displayed electric-driven release behavior, releasing drugs more rapidly when subjected to voltage, demonstrating their potential as electrically responsive DDSs. Applying a 3 V voltage significantly increased the release of dexamethasone and indomethacin from the hydrogel matrices compared to no voltage (Qu et al., 2019).

The biocompatibility of Dex-AT/HDI hydrogels was assessed through staining after subcutaneous implantation in rats. Initially, hydrogel samples showed mild inflammatory responses with no significant differences in the number of inflammatory cells. After 28 days, the inflammatory responses decreased significantly in all groups, and toluidine blue staining revealed equivalent mast cell counts. These findings indicate good biocompatibility, making these hydrogels promising for *in vivo* drug delivery applications (Qu et al., 2019).

Gangrade et al. developed a photo-electro-responsive nanocomposite silk-based hydrogel for on-demand drug release (Gangrade et al., 2020). Single-wall carbon nanotubes (SWCNTs) were cross-linked with folic acid to form SWCNT-FA, and DOX was adsorbed onto SWCNT-FA. Silk hydrogel was created by combining two silk fibroin (SF) proteins, and SWCNT-FA/DOX was incorporated into the blend. The resulting nanocomposite silk/SWCNT-FA/DOX hydrogel transitioned from solution to gel at 37°C (Gangrade et al., 2020).

Silk/SWCNT-FA/DOX hydrogel demonstrated electroactivity and suitability for electrically stimulated drug release. The hydrogel exhibited nonlinear behavior in current-voltage characteristics, indicating possible Ohmic and space charge-limited conduction. Electric field-triggered drug release was tested. The hydrogel showed a controlled release of DOX with multiple stimulations. Silk/SWCNT-FA/DOX hydrogel outperformed silk/DOX hydrogel in drug release, showcasing its potential for on-demand drug delivery. A theory was proposed to explain the release mechanism involving ion movement and electrostatic interactions.

Additionally, the photothermal action of prepared hydrogel under NIR laser exposure was investigated. The hydrogel responded rapidly to the NIR laser, inducing a temperature increase. The drug release from silk/SWCNT-FA/DOX hydrogel post-NIR radiation was also observed, and the release mechanism was explained based on the hydrogel’s shrinkage and structural changes induced by heat (Gangrade et al., 2020).

In an *in vivo* study on tumor regression, silk/SWCNT-FA/DOX was tested in mice with breast solid tumors. Groups receiving this treatment along with external stimulation (NIR laser, electric field, or both) showed significant tumor regression, while untreated and systemically treated groups showed tumor growth. Western blotting suggested apoptosis induction in treated groups. Tunnel assay and histological analysis supported these findings. Notably, there were no signs of cardiotoxicity, likely due to the low DOX dose (Gangrade et al., 2020). Unfortunately, it is not convenient to use electric-driven hydrogels in practice due to wire-connected bulky equipment.

### 3.9 Magnetic-responsive hydrogels based on natural polymers

**FIGURE 6:**
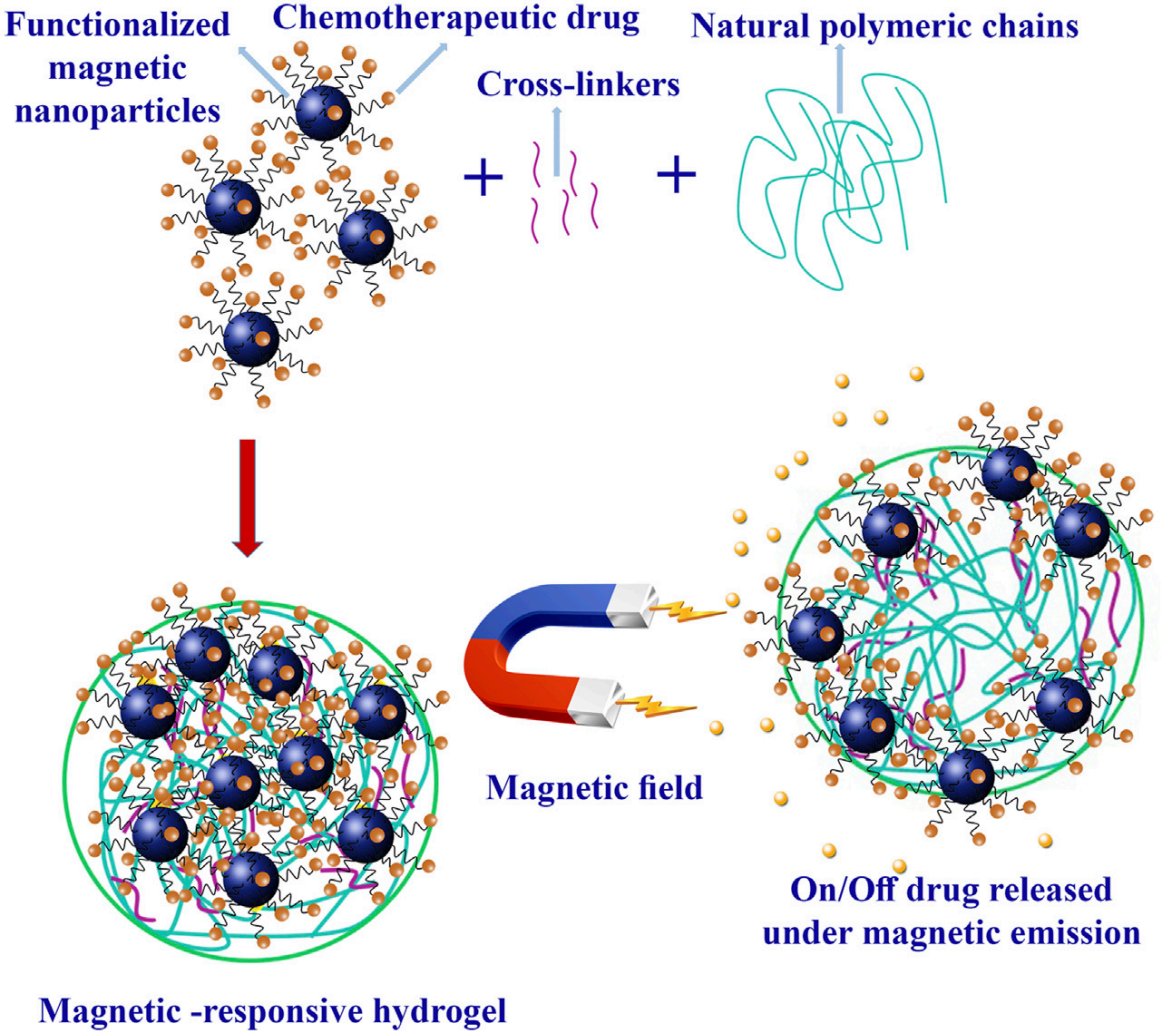
Magnetic hydrogel preparation and chemotherapeutic drug released under magnetic emissions.

Magnetic-responsive hydrogels harness magnetic fields to modulate their properties. These hydrogels typically incorporate iron oxide NPs with paramagnetic properties, which vibrate when exposed to a magnetic field, leading to a localized temperature increase (Kasiński et al., 2020). This property can be exploited for thermal-ablation mechanisms, enhancing therapeutic efficacy. This approach combines thermal and chemotherapeutic cytotoxicity synergistically with thermo-sensitive hydrogels, where temperature changes trigger drug release (Figure 6). It offers spatiotemporal control, non-invasive and deep tissue penetration (Gao et al., 2019a).

Gao et al. prepared a magnetic hydrogel functionalized by ferromagnetic vortex-domain iron oxides (FVIOs) to prevent breast cancer recurrence. FVIOs were synthesized from α-Fe_2_O_3_ nanorings via thermal transformation. The coating of oleic acid (OA) was achieved by mixing trioctylamine and OA, followed by heating and purification. OA-modified superparamagnetic iron oxide nanoparticles (SPIONs) were synthesized through the thermal decomposition of an iron-oleate complex. Aqueous phase transfer of FVIOs and SPIONs was performed with 3,4-dihydroxy-hydrocinnamic acid (DHCA). To prepare magnetic hydrogels, CS-FVIOs, and CS-SPIONs solutions were synthesized by blending DHCA-modified particles with CS solutions. Drug-loaded magnetic hydrogels were formed by mixing DOX in a DF solution with the glycol-CS ferrofluid. Control hydrogels were also prepared without FVIOs, SPIONs, or DOX (Gao et al., 2019b).

The magnetic nanoparticle dopant was crucial in assessing the temperature elevation of the magnetic hydrogel. FVIOs were chosen for their effective heat induction due to their unique magnetic reversal process. FVIO-functionalized magnetic hydrogel (F-MH) showed injectability, self-healing, and the ability to conform to its surroundings. The hydrogel released more dye at a lower pH, indicating acid responsiveness. The unique properties of F-MH were attributed to the balance between poly (ethylene glycol) and CS chains (Li et al., 2015). The induction heat capabilities of the F-MH revealed a dependency on nanoparticle dopants and dose. F-MH and SPION-functionalized magnetic hydrogels (S-MH) presented improved heating under a high-intensity AMF, but F-MH exhibited superior heating capacity. F-MH required much lower Fe concentrations to achieve equivalent temperature increases than S-MH. The specific absorption rate (SAR) values for F-MH were significantly higher, indicating better induction heat performance. S-MH showed delayed gelation, decreased adherence, and lower stability than F-MH. Biodegradation tests showed that F-MH maintained stability even under acidic conditions, suggesting its suitability for sustained synergistic thermo-chemotherapy. *In vivo* tests demonstrated F-MH’s prolonged stability and potential for long-term therapeutic efficacy. *In vitro* drug release tests utilized DOX-loaded F-MH. DOX release rates were higher at an acidic pH, similar to the microenvironment in tumors. *In vivo* experiments on tumor-bearing mice confirmed selective DOX release in tumor tissues due to F-MH’s design. The combined effect of DOX and AMF irradiation significantly decreased breast cancer cell viability, and fluorescence imaging revealed enhanced DOX delivery into cell nuclei, indicating successful synergistic thermo-chemotherapy (Gao et al., 2019b).

*In vivo*, evaluations were conducted on tumor-bearing mice. After surgical resection, the hydrogel was administered into the residual tumor bed. AMF exposure elevated tumor surface temperatures in the F-MH and DOX-F-MH groups, leading to necrosis and tumor shrinkage. Over 90% of tumor shrinkage and prolonged lifespan were seen in the DOX-F-MH group. Histological assessments showed no systemic toxicity (Gao et al., 2019b).

Wu et al. prepared injectable magnetic hydrogel and evaluated its therapeutic applications in preventing the postoperative recurrence of breast cancer (Wu et al., 2018). Oleic acid-coated Fe_3_O_4_ NPs were synthesized through thermal decomposition and then made water-soluble (Wu et al., 2018). These NPs were combined with α-cyclodextrin DOX and paclitaxel (PTX) to create magnetic responsive hydrogels with adjustable properties. To prepare magnetic gellan gum hydrogels, gellan gum, sodium, and 1,2-propanediol propionate were dissolved in deionized water while stirring at 90°C. Then, the mixture was sonicated in hot water. Afterward, an aqueous solution of PEGylated Fe3O4 NPs, maintaining an iron concentration of 3 mg mL^−1^, was added to the gel (Wu et al., 2018).

The hydrogels had injectable structures with shear-thinning properties for pattern creation. PEGylated Fe3O4 NPs showed a lipid layer and a magnetic core. The hydrogel released PTX and DOX due to its hierarchical structure. PTX released slowly, and DOX had an initial rapid release followed by sustained release. Under AMF irradiation, the hydrogel accelerated drug release through increased local temperature. Fe_3_O_4_ NPs generate heat under an AMF, leading to gel-sol transition in the hydrogel. The critical temperature can be adjusted via polyethylene glycol and α-cyclodextrin concentrations (Wu et al., 2018). Magnetic hydrogels with a critical temperature of around 45°C can be used for safer thermo-chemotherapy (Wu et al., 2017).

Moreover, mild AMF is safe for potential biomedical use (Xie et al., 2014). In cancer treatment, irregular postoperative cavities pose challenges. This hydrogel could have been injected into tumor sites and, under AMF irradiation, adapted to irregular shapes in mice (Wu et al., 2018).

In the *in vivo* evaluations in mice, they resected breast tumors and administered the hydrogel. Gellan-gum magnetic hydrogel effectively released drugs over 15 days and eroded gradually. Histological analysis demonstrated tumor regression, wound healing, and organ safety. It also prevented recurrence and exhibited high survival rates (Wu et al., 2018).

Eivazzadeh-Keihan et al. synthesized a magnetic nano biocomposite to manage hyperthermia cancer treatment (Eivazzadeh-Keihan et al., 2022). The cross-linked sodium Alg hydrogel was prepared by dissolving SA powder in distilled water and adding CaCl_2_ as a cross-linker. SF was extracted, and dialysis for the cross-linked SA hydrogel/SF composite. SF was mixed with cross-linked SA hydrogel at a 1:1 ratio. Halloysite nanotubes (HNTs) were added to the cross-linked SA hydrogel/SF composite to strengthen the structure and sonicated for uniform dispersion. The final cross-linked Alg hydrogel/SF/HNTs/Fe3O4 biocomposite scaffold was synthesized by adding FeCl_3_.6H_2_O and FeCl_2_.4H_2_O to the mixture, heating, and stirring under N2 atmosphere, followed by ammonia addition and purification to achieve a neutral pH (Eivazzadeh-Keihan et al., 2022). The assessment of thermal stability revealed distinct weight loss stages. The initial 10% mass loss was attributed to moisture desorption—incorporating organic species with HNTs reduced moisture loss and enhanced HNTs’ hydrophobicity. The second weight loss was linked to the decomposition of SF’s amino acid side chains and peptide bonds. The third mass loss was associated with the degradation of the Alg. The bio composite’s magnetic properties were investigated using a vibrating-sample magnetometer, with a magnetization of approximately 15.96 emu g−1 due to its core-shell structure. Biological assessments included a red blood cell lysis inhibition assay, demonstrating that the biocomposite exhibited minimal hemolysis and was fully compatible with blood. Cell proliferation assays indicated that the synthesized biocomposite was non-toxic to normal cells over 48 and 72 h, while it reduced the proliferation rate and viability of breast cancer cells (Eivazzadeh-Keihan et al., 2022).

The biocomposite’s application was evaluated for hyperthermia, a cancer therapy method that elevates tumor temperature. The biocomposite maintained structural integrity at elevated temperatures. The rate of temperature increase was affected by MNP concentration and frequency. The highest SAR was achieved at the highest concentration and frequency (Eivazzadeh-Keihan et al., 2022).

A significant obstacle in utilizing magnetic-responsive hydrogels is the compatibility and degradation of the magnetic additives.

## 4 Multi-responsive

**FIGURE 7:**
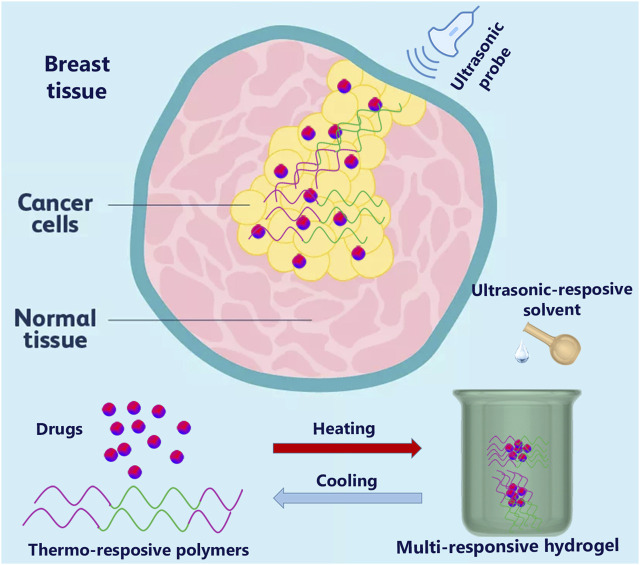
The multi-responsive hydrogel based on temperature and ultrasound triggers for breast cancer.

Combining two or more stimuli units into one hydrogel system has recently become a research hotspot with broad application prospects. Multi-stimuli-responsive hydrogels based on natural polymers have emerged as a new trend in achieving specified theranostics hydrogel systems and reducing complications for breast cancer treatments (Figure 7) (Li et al., 2022). However, despite the difficulties associated with synthesizing multiple stimuli-responsive hydrogels, such as being time-consuming and requiring various steps, they showed more progress than mono-responsive hydrogels. The arrangement of distinct gelators and cross-links in multi-stimuli-responsive hydrogels facilitates their functions (Xia et al., 2021). The stimuli-responsive hydrogel based on natural polymers may provide a rich “toolbox” for tailoring intelligent materials (Xu et al., 2017).

Schmidt et al. created dual (light and temperature) stimuli-responsive HA and poly (N-isopropyl acrylamide) (PNIPAM) microgels for breast cancer cell diagnostic and therapeutic procedures. The microgels were fabricated using the precipitation polymerization technique with amine-bearing co-monomers, and HA was functionalized with a UV-cleavable linker. The dual stimulus-responsive microgels revealed significantly adherent CD44-expressing breast cancerous cells (MDA-MB-231 and MCF-7), which increased with higher levels of HA functionalization. At temperatures below the LCST, the thermos-responsive properties of microgels lead to the cells being released; however, 10%–30% of the cells remain on the hydrogel. Complete cell release was observed after short UV exposure, cleaving HA photoactive cross-linker units from the dual stimuli-responsive cells. The result showed that smart microgel could be a promising candidate for breast cancer cell treatment (Schmidt et al., 2021).

Cao et al. developed novel and effective multi-responsive (light, magnetic, and pH) GOFe3O4/PNIPAM/Alg nanocomposite hydrogel microcapsules for chemotherapeutic drug release. The GO−Fe3O4 nanomaterials, upon NIR light radiation and changing magnetic force, increased temperature above the LCST and caused the release of DOX through deswelling. Furthermore, combining Alg with PNIPAM polymer improved the gelation process, mechanical properties, and pH-responsive performance. The *in vitro* cytotoxicity assay demonstrated that a multi-responsive hydrogel platform can effectively reduce the cell viability of MCF-7 (Cao et al., 2021).

Derakhshankhah et al. developed a pH and thermal-responsive magnetic hydrogel based on a natural gel polymer for cancer chemo/hyperthermia therapy. 3-(trimethoxysilyl) propyl methacrylate (MPS)- Fe_3_O_4_ NPs were synthesized using co-precipitation (Derakhshankhah et al., 2021). The SEM images demonstrated porous structures in the hydrogels, leading to high drug loading and encapsulation efficiencies. The strong physical interactions between DOX and hydrogel functional groups contributed to these high efficiencies. *In vitro* drug release studies showed that the developed hydrogel exhibited pH- and temperature-dependent release behaviors. Hydrogel showed increased drug release due to the effect of temperature-responsive PNIPAAm chains. Kinetic analysis indicated that drug release from prepared magnetic hydrogel follows a diffusion mechanism, best represented by the Higuchi square root model (Derakhshankhah et al., 2021).

Xie et al. presented an injectable thermos and magnetic responsive CS hydrogel that contained DOX and docetaxel (DTX) for chemotherapy and iron oxide for magnetic hyperthermia-induced drug release. Docetaxel-loaded PLGA NPs (DTX/PLGA) were created using solvent extraction/evaporation. Iron oxide magnetic NPs (Fe_3_O_4_/DF-PEG-DF MNPs) modified with difunctional telechelic polyethylene glycol (DF) were synthesized through a hydrothermal approach. The final step included combining DOX, DTX/PLGA NPs, and Fe_3_O_4_/DF-PEG-DF with a CS solution, then adding DF and forming the hydrogel (Xie et al., 2017).

The hydrogel showed the ability to heal itself after experiencing structural damage. Toxicity tests on mice indicated no significant adverse effects on organs or blood parameters. *In vitro* drug release studies revealed that the hydrogel exhibited sustained drug release over time. Interestingly, the co-delivery of DOX and DTX led to a mutually enhanced release of both drugs. The magnetic properties of the hydrogel enabled controlled temperature changes through an alternating magnetic field (AMF), which accelerated the release of DTX due to changes in the hydrogel’s structure (Xie et al., 2017).

*In vivo*, tests on tumor-bearing mice showed that the hydrogel exhibited effective anti-tumor effects. Injecting it into tumors and subjecting them to an AMF raised their temperature significantly, enhancing drug release. The co-delivery of DOX and DTX/PLGA NPs resulted in synergistic anti-tumor effects, outperforming single-drug treatments. Mice treated with the fabricated hydrogel and exposed to AMF demonstrated remarkable anti-tumor activity within 4 days, with minimal systemic toxicity indicated by stable body weight (Xie et al., 2017).

In another study, Gao et al. evaluated the inductive heating property of hypertonic saline (HTS) under AMF to prevent breast cancer recurrence. HTS-PEG hydrogel was prepared by mixing glycol CS and DF solutions with sodium chloride. A control hydrogel was also prepared without the saline solution (Gao et al., 2019c).

HTS solutions showed temperature increases under AMF exposure, with higher field intensity and HTS concentration leading to higher temperature elevations. Dielectric constant (ε′) measurements revealed a positive correlation between ε′ and inductive heating efficiency. The mechanism involves the interaction of charged ions with electromagnetic radiation, generating heat through dipolar polarization (Menéndez et al., 2010). The HTS-PEG hydrogel effectively confined ions, leading to reproducible heating responses. The hydrogel’s confining capacity was attributed to its network’s homogeneity. The findings suggest potential applications for controlled heating in clinical settings, particularly for hyperthermia treatment of tumors (Gao et al., 2019c).

An *in vivo* study evaluated the anti-tumor efficacy of DOX-HTS-PEG hydrogel for postsurgical recurrence prevention using breast tumor models in mice. The DOX-HTS-PEG hydrogel + AMF showed complete tumor elimination due to combined chemotherapy and hyperthermia effects. Organ histology and body weight remained unaffected (Gao et al., 2019c).

## 5 Regulatory approval and clinical translation

Despite the advancement of smart hydrogels based on natural polymers in the research domain, it is hindered by several technological hurdles, such as chemistry, good manufacturing practices (cGMPs), quality controls, scale-up, and established regulatory guidelines, that prevent their successful clinical translation. The natural polymers present excellent biocompatibility and biodegradability and eliminate second surgery for implant removal. However, risks of low harvested quantities, batch-to-batch variation, and complex purification processes threaten long-term safety and scalability areas. Furthermore, stimuli-responsive hydrogels’ swelling and sensitive construction necessitated intricate sterilization, preservation, and production technologies. Fabricating stimuli-responsive hydrogels based on natural polymers’ is complex, requiring a variety of cross-linking agents and biomaterials, which enhances their safety and regulatory approval from authorities. Stimuli-responsive hydrogels based on natural polymer categories as “devices” and the production of their products for clinical uses may require estimates ranging from $10 million to $500 million (Mandal et al., 2020; Cao et al., 2023).

Natural polymer hydrogels may have more drawbacks in their heterogeneity, robustness, solubility, and inflammatory complications when they are implanted into the body. Furthermore, the breast tissues are mostly under external stresses and receive weak support from the surrounding protective tissues such as muscles, ligaments, and skin. Sleeping, exercising, physical movements, and other pressures might cause hydrogel displacements, pain, alteration in thermoregulation, and other complications for patients. Thus, improving the mechanical properties, stability, biodegradability, biocompatibility, and purity of natural hydrogels a cross with the realistic ECM models hydrogel with a complex of fibrous, granular, colloidal structures and stimuli-responsive features might hold an alternative potential as breast cancer diagnostic and treatment tools.

Recently, the safety and quality of cross-linkers, polymers, and other reagents significantly increased. The new progress in nanomaterials, cross-linker agents, polymer chemistry, and fabrication technology standardization helped improve the rheological and mechanical properties of hydrogels based on natural polymers accommodated with breast tissue structure. HA, CS, Alg, Gel, fibrin, and hydroxypropyl methylcellulose (HPMC) have been extensively explored due to their compatibility with natural biological matrices (Mandal et al., 2020; Mohapatra et al., 2021)—several examples of clinical trials conducted based on natural polymer hydrogels in breast cancer (Table 2). Among natural hydrogels, HA attracted bioinspiration potential in breast cancer treatments and cosmetic implant applications and was preferred to collagen-based products for its lower immunogenicity and more extended durability. The HA is the most interesting and effective natural filler for breast cosmetics and augmentation in aesthetic surgery, which is frequently administrated to customers. Several HA gel-based fillers approved by the FDA and European medicine agencies are commercially available. However, limited complications associated with HA gel administration in breast tissue were reported (Trignano et al., 2020). HA is an excellent natural polymer component of connective tissues such as skin, cartilage, and synovial fluids.

**TABLE 2:** Current clinical trial studies based on natural polymer hydrogels in breast cancer.

Condition	Polymer	Result posted	Number	Recruitment status	Location
Radiation-Induced Dermatitis	HA	No	NCT04995328	Completed	Taichung, Taiwan, China Medical University Hospital
Vaginal and Sexual Dysfunction After Breast Cancer Treatment		No	NCT04713917	Recruiting	University Hospital of Henri Mondor and the University of Paris Est Creteil
					France
Non-Hormonal Vaginal Moisturizer in Hormone-Receptor Positive Postmenopausal Breast Cancer Survivors		No	NCT01738152	Completed	Memorial Sloan Kettering Cancer Center, New York, United States
Breast cancer wounds	Alginate	No	NCT05800834	Recruiting	Daianny Arrais de Oliveira da Cunha, Instituto Nacional de Cancer, Brazil
Vulvovaginal Atrophy Management in Breast Cancer Patients	Collagen	No	NCT05585476	Not yet recruiting	Institute of Health Research at Jiménez Díaz Foundation (IIS-FJD), Spain
Reduction of Post-surgical Serous Drainage		No	NCT04904653	Recruiting	Instituto de Investigación Hospital Universitario La Paz, Spain
Advanced Solid Tumors	Gelatin	yes	NCT04672460	Completed	Pfizer, United States

Furthermore, HA could hydrate and help to preserve the natural shapes of breast tissues. HA decorated nanoparticles showed significant interaction with breast cancerous cells and could suppress the metastasis cells. Regarding the investigation in clinical and experimental literature, HA presented the greatest potential in developing intelligent hydrogels based on natural polymers for enhanced breast cancer care (Alsaikhan, 2023).

## 6 Conclusion and prospects

This review encompasses stimuli-responsive hydrogels based on natural polymer biomedical applications in breast cancer. Natural polymers have been demonstrated to be efficacious in various stimulus-sensitive applications. Modifying hydrogel backbones with biomaterials and functional groups for a stimuli-responsive state improves their mechanical and biocompatible properties. Stimuli-responsive hydrogels with single, dual, or multi-responsive features possess great potential for the controlled release of chemotherapeutic agents. Stimuli-responsive hydrogels derived from natural polymers, which are biodegradable, non-toxic, and highly biomimetic, will possess a broader potential to be utilized in breast cancer. Research about stimuli-responsive natural polymer-based hydrogels will likely be a hot topic in the coming decade.
